# Cystic fibrosis-related mortality in the United States from 1999 to 2020: an observational analysis of time trends and disparities

**DOI:** 10.1038/s41598-023-41868-x

**Published:** 2023-09-12

**Authors:** Harpreet Singh, Chinmay Jani, Dominic C. Marshall, Rose Franco, Padmanabh Bhatt, Shreya Podder, Joseph Shalhoub, Jonathan S. Kurman, Rahul Nanchal, Ahmet Z. Uluer, Justin D. Salciccioli

**Affiliations:** 1 Division of Pulmonary and Critical Care Medicine, Medical College of Wisconsin, 8701 Watertown Plank Road, Milwaukee, WI 53226 USA; 2 Division of Pulmonary and Critical Care, Brigham and Women’s Hospital, Boston, MA USA; 3 Division of Pulmonary Medicine, Boston Children’s Hospital, Boston, MA USA; 4 National Heart and Lung Institute, Imperial College London, London, UK; 5 Department of Medicine, Mount Auburn Hospital/Beth Israel Lahey Health, Cambridge, MA USA; 6 Harvard Medical School, Boston, MA USA; 7 Imperial College Healthcare NHS Trust, London, UK; 8 Medical Data Research Collaborative, London, UK; 9 Department of Surgery and Cancer, Imperial College London, London, UK

**Keywords:** Epidemiology, Respiratory tract diseases, Cystic fibrosis

## Abstract

Cystic fibrosis transmembrane conductance regulator modulators have revolutionized cystic fibrosis (CF) care in the past decade. This study explores the CF-related mortality trends in the US from 1999 to 2020. We extracted CF-related mortality data from the CDC WONDER database. CF age-standardized mortality rates (ASMRs) were identified by ICD-10 code E84 and were stratified by demographic and geographical variables. Temporal trends were analyzed using Joinpoint modeling. CF-related ASMRs decreased from 1.9 to 1.04 per million population (*p* = 0.013), with a greater reduction in recent years. This trend was replicated in both sexes. The median age of death increased from 24 to 37 years. CF mortality rates decreased across sex, white race, non-Hispanic ethnicity, census regions, and urbanization status. Incongruent trends were reported in non-white races and Hispanic ethnicity. A lower median age of death was observed in women, non-white races, and Hispanic ethnicity. SARS-CoV-2 infection was the primary cause of death in 1.7% of CF decedents in 2020. The national CF-related mortality rates declined and the median age of death among CF decedents increased significantly indicating better survival in the recent years. The changes were relatively slow during the earlier period of the study, followed by a greater decline lately. We observed patterns of sex, ethnic, racial, and geographical disparities associated with the worsening of the gap between ethnicities, narrowing of the gap between races and rural vs. urban counties, and closing of the gap between sexes over the study period.

## Introduction

Cystic fibrosis (CF) is an inherited multi-system disease with a shortened life expectancy, affecting approximately 40,000 people in the United States (US) and 105,000 people worldwide^1^. Although CF is most common in Caucasians, affecting 1 in 2500 live births, it is being increasingly recognized in other races and ethnicities including 1 in 10,500 Native Americans, 1 in 13,500 Hispanic-Americans, 1 in 15,000 African-Americans, and 1 in 35,000 Asian-Americans^2,3^. Cystic fibrosis is an autosomal recessive disease caused by mutations in the cystic fibrosis transmembrane conductance regulator protein (CFTR), resulting in defective chloride and bicarbonate secretion in epithelial cells, with greater than 2000 CFTR mutations identified to date^4^. While CFTR dysfunction is genetically influenced by the mutation class, often leading to life-threatening complications^5^, the F508del mutation accounts for the majority of mutated alleles in North American and northern European populations.

Cystic fibrosis care has evolved steadily over the last 70-plus years, most dramatically in the past decade, with advances in diagnostic and therapeutic options including nationwide newborn screening to identify CF at birth, aggressive infection prevention, targeted nutritional support, augmentation of pancreatic function, improved mucociliary clearance, and the implementation of highly effective CFTR modulators^6^. Several recent studies have reported encouraging survival trends^7–10^. To date, the available studies from the US utilized data from the Cystic Fibrosis Foundation Patient Registry (CFFPR), composed of patients cared for at specialized CF centers. The US CFFPR is a comprehensive registry that records 81–84% of CF patients with a 90% retention rate, capturing close to 72–75% of CF patients for clinical research^11^. However, no recent US study has reported CF-related mortality trends incorporating the decedents who did not participate in CFFPR. The Centers for Disease Control and Prevention (CDC) Wide-ranging Online Data for Epidemiologic Research (WONDER) Multiple Cause of Death (MCOD) data files, which cover the death records of the whole US population, provide a unique opportunity to analyze the evolution of CF mortality independent of the decedents' participation in the CFFPR.

In this study, we aimed to investigate temporal trends of CF-related mortality in the US. We also explored disparities by demographic characteristics and geographical regions.

## Methods

### Study design

This is a retrospective observational study performed using CDC WONDER MCOD datafiles from 1999 to 2020. MCOD data files are compiled by the Division of Vital Statistics, the National Center for Health Statistics (NCHS), and the United States Department of Health and Human Services. The death certificate of US residents is the primary source of data**.** The method of translation of death certificate data into MCOD files has been reported previously^12^. MCOD files include an underlying cause of death (UCOD), up to 20 additional contributing causes of death, and demographic information about the decedents. The UCOD is defined as “the disease or injury which initiated the train of events leading directly to the death". Deidentified data was publicly available for researchers and an institutional review board endorsement was not required.

### Study definitions

CF-related deaths were identified by the International Classification of Diseases-10 (ICD-10) code E84. Age-standardized mortality rates (ASMRs) were stratified by sex, race, ethnicity, census region, urbanization, and age. For race-specific estimates, we reported four race categories: White, African American, American Indians/Alaska Natives, and Asian/Pacific Islanders. For urbanization-specific data, the analysis was reported based on counties’ population sizes > 250 K, and < 250 K. In addition, we calculated the median age of death in the years 1999, 2005, 2010, 2015, and 2020 for the CF population and at the start and end of the study period for each sex, race, and ethnicity to make our analysis comparable to previously published literature.

### Statistical analysis

CF-related ASMRs with a 95% confidence interval (CI) were extracted. The use of age standardization is common in epidemiological studies to provide information regarding changes in mortality trends over time as well as for making comparisons between demographic variables. For this analysis, we used the 2000 US standard population as provided by the CDC, and mortality rates were computed as deaths per million population. The extracted ASMRs were analyzed using Joinpoint regression modeling (Command Line Version 4.5.0.1), a statistical software developed by the US National Cancer Institute Surveillance Research Program to identify any changes in mortality trends and time points with significant inflections^13,14^. The average annual percentage changes (AAPCs) with CI were reported. Additionally, the model computes an estimated annual percentage changes (EAPCs) for each trend segment by fitting a regression line to the natural logarithm of ASMRs. Null hypothesis testing of whether EAPCs or AAPCs were non-zero was performed. Per CDC protocol to ensure confidentiality, individual mortality trends for each non-white race were not reported due to < 10 deaths per annum and automated suppression of MCOD datafiles. We re-analyzed the CF mortality trends by using UCOD as the case definition to record any disparities based on the alternate case definition. UCOD CF mortality rates were stratified similarly to the primary analysis, but Joinpoint analysis was performed on overall national-level data only.

### Sensitivity analysis

After completing the primary analysis, we planned two separate post hoc sensitivity analyses to assess the potential influence of the severe acute respiratory syndrome coronavirus 2 (SARS-CoV-2) pandemic on the observed trends. First, we extended the observation period to include provisional mortality statistics for the year 2021 which are also provided by the CDC. Briefly, the provisional mortality data reports all the variables similar to the final MCOD files. The only difference is provisional data might not include all the deaths. However, this entry process lags by just a few weeks making 2021 data equally reliable. We graphically inspected the mortality trends and repeated the Joinpoint analysis including the additional year of data. Second, if the observed trends in CF mortality were due to changes that came with the SARS-CoV-2 pandemic, it is possible that mortality from other chronic respiratory diseases would follow a similar trend after the onset of the pandemic. Therefore, we analyzed mortality data of other chronic respiratory diseases (ICD-10 codes: J40-J47; J84).

## Results

From 1999 to 2020, 56,806,341 individuals died in the US, and CF accounted for 11,068 deaths (0.02%) including 5489 (49.6%) men, and 5579 (50.4%) women. The corresponding average CF-related ASMR was 1.65 per million population (95% CI 1.61–1.68). Individually in men and women, average CF-related ASMRs were 1.67 (CI 1.62–1.71) and 1.68 (CI 1.64–1.72) respectively. The highest number of deaths (n = 10479, [95%]) were recorded in Whites with ASMR of 2.01 (CI 1.97–2.05), followed by 461 (4%) in African American (ASMR, 0.48 [CI 0.43–0.52)]), 68 (0.5%) in American Indians/Alaska Native (ASMR, 0.75(CI 0.57–0.96]), and 60 (0.5%) in Asian/Pacific Islanders with ASMR of 0.17 (CI 0.13–0.23). By ethnicity, the majority of deaths [n = 10333, (93%)] occurred in non-Hispanic ethnicity with ASMR of 1.87 (CI 1.84–1.91) in comparison to Hispanic (n = 722 [7%]; ASMR, 0.64 [CI 0.59–0.69]) (Table 1).

**Table 1:** Cystic fibrosis-related average age-standardized mortality rates per million population by demographic characteristics, and geographical regions in the United States, 1999–2020.

Variables	Deaths	Population	Average ASMRs	(95% CI) LL	(95% CI) UL
US overall	11,068	6,746,356,647	1.65	1.61	1.68
Gender					
Men	5489	3,317,352,843	1.67	1.62	1.71
Women	5579	3,429,003,804	1.68	1.64	1.72
Race					
White	10,479	5,367,045,067	2.01	1.97	2.05
Men	5182	2,654,949,252	1.99	1.93	2.04
Women	5297	2,712,095,815	2.08	2.02	2.13
African American	461	919,034,937	0.48	0.43	0.52
Men	240	439,446,295	0.44	0.39	0.5
Women	221	479,588,642	0.54	0.47	0.61
American Indians/Alaska Native	68	88,362,592	0.75	0.57	0.96
Men	35	44,272,656	0.73	0.5	1.03
Women	33	44,089,936	0.73	0.5	1.04
Asian/Pacific Islanders	60	371,914,051	0.17	0.13	0.23
Men	32	178,684,640	0.2	0.14	0.29
Women	28	193,229,411	0.14	0.09	0.21
Ethnicity					
Non-Hispanic	10,333	5,669,076,309	1.87	1.84	1.91
Men	5139	2,769,815,073	1.88	1.83	1.94
Women	5194	2,899,261,236	1.91	1.86	1.96
Hispanic	722	1,077,280,338	0.64	0.59	0.69
Men	345	547,537,770	0.61	0.58	0.69
Women	377	529,742,568	0.65	0.58	0.72
Census regions					
Northeast	2007	1,212,994,922	1.72	1.64	1.79
Midwest	2602	1,466,121,214	1.82	1.75	1.89
South	4215	2,497,818,081	1.73	1.68	1.78
West	2244	1,569,422,430	1.44	1.38	1.5
Urbanization					
Counties with population > 250 K	7773	5,119,450,801	1.53	1.5	1.56
Counties with population < 250 K	3295	1,626,896,500	2.13	2.06	2.2

*ASMRs* Age-standardized mortality rates, *CI* Confidence interval, *LL* Lower limit, *US* United States, *UL* Upper limit.

*Urbanization > 250 K = large to medium metropolitans; < 250 K = small metro to non-metro.

Data from multiple cause of death CDC WONDER data set, 1999–2020.

The CF mortality rates varied across the census regions with the highest average ASMR in the Midwest (1.82 per million population [CI 1.75–1.89]) and lowest in the West (1.44, CI [1.38–1.5]). CF mortality burden was lower in counties with a population > 250 K (1.53 [CI 1.50–1.56]) versus counties < 250 K (2.13 [CI 2.06–2.20]) (Table 1).

**Figure 1:**
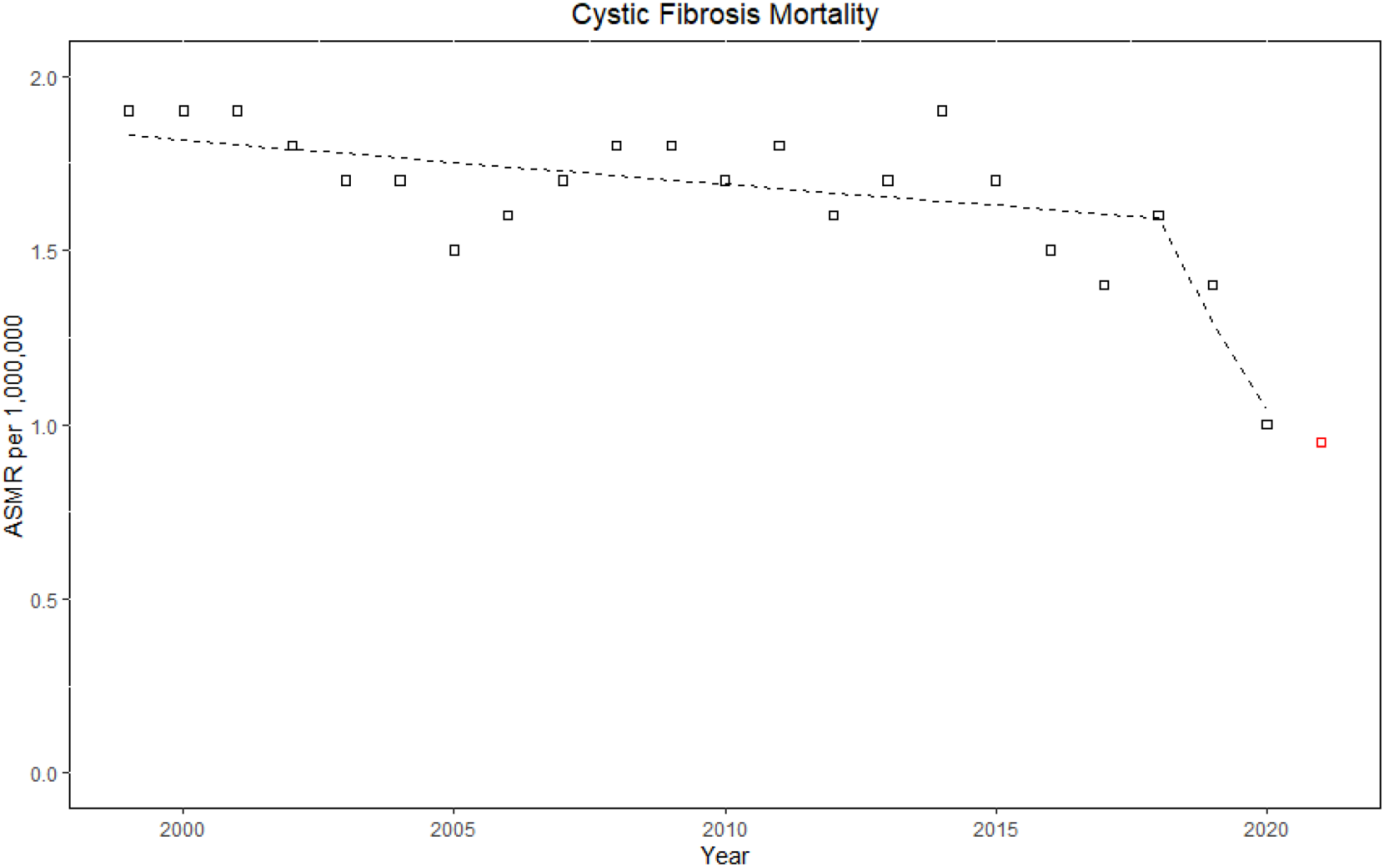
Joinpoint trends of cystic fibrosis-related age-standardized mortality rates per million population in the United States, 1999–2020. The red square indicates CF-ASMR in the year 2021 using provisional CDC WONDER data files.

Table 2 and Fig. S1 report the CF mortality rates analyzed by age group and sex (Fig. 1). Women CF patients reached higher and earlier peak mortality (15–24 age group) as compared to men (25–34 age group). The highest number of deaths was identified in the 5–24 age group (n = 3212 [29%]), and the lowest number of deaths was estimated in the 85 + age group (n = 105 [0.9%]). The corresponding ASMR was highest in the 15–24 age group (3.43 per million population [CI 3.31–3.55]), and lowest in the 1–4 years age group (0.31 [CI 0.25–0.37]).

**Table 2:** Cystic fibrosis-related crude mortality rates per million population by age groups, and sex in the United States, 1999–2020.

Age	Men			Women			Overall		
	Deaths	Population	Average CMRs (95% CI)	Deaths	Population	Average CMRs (95% CI)	Deaths	Population	Average CMP. (95% CI)
< 1 year	127	44,436,653	2.86 (2.36–3.36)	34	42,477,103	2.21 (1.79–2.71)	221	86,913,756	2.54 (2.21–2.88)
1–4 years	51	178,140,207	0.29 (0.21–0.38)	58	170,430,172	0.34 (0,26–0.44)	109	348,570,379	0.31 (0.25–0.37)
5–14 yea rs	275	460,784,106	0.6 (0.53–0.67)	374	440,439,195	0.85 (0.76–0.94)	649	901,223,301	0.72 (0.66–0.78)
15–24 years	1430	479,434,966	2.98 (2.83–3.14)	1782	456,359,756	3.9 (3.72–4.09)	3212	935,794,722	3.43 (3.31–3.55)
25–34 years	1581	464,158,283	3.41 (3.24–3.57)	1494	455,931,186	3.28 (3.11–3.44)	3075	920,089,469	3.34 (3.22–3.46))
35–44 years	923	463,579,553	1.99 (1.86–2.12)	794	467,707,735	1.7 (1.58–1.82)	1717	931,287,288	1.84 (1.76–1.93)
45–54 years	534	456,507,175	1.17(1.07–1.27)	419	471,069,045	0.89 (0.8–0.97)	953	927,576,220	1.03 (0.96–1.09)
55–54 years	288	369,467,361	0.78 (0.69–0.87)	242	396,957,486	0.61 (0.53–0.69)	530	766,424,847	0.69 (0.63–0.75)
65–74 years	142	236,529,573	0.6 (0.5–0.7)	157	273,928,768	0.57 (0.48–0.66)	299	510,458,341	0.59 (0.52–0.65)
75–84 years	07	125,077,029	0.78(0.63–0.95)	101	173,427,404	0.58 (0.47–0.7)	198	298,504,433	0.66 (0.57–0.76)
85 + years	41	39,237.937	1.04 (0.75–1.42)	64	80,275,954	0.8 (0.61–1.02)	105	119,513,891	0.88 (0.71–1.05)

*CI* confidence interval, *CMRs* Crude mortality rates, *US* United States.

Data from multiple came of death CDC WONDER data set, 1999–2020.

**Figure 2:**
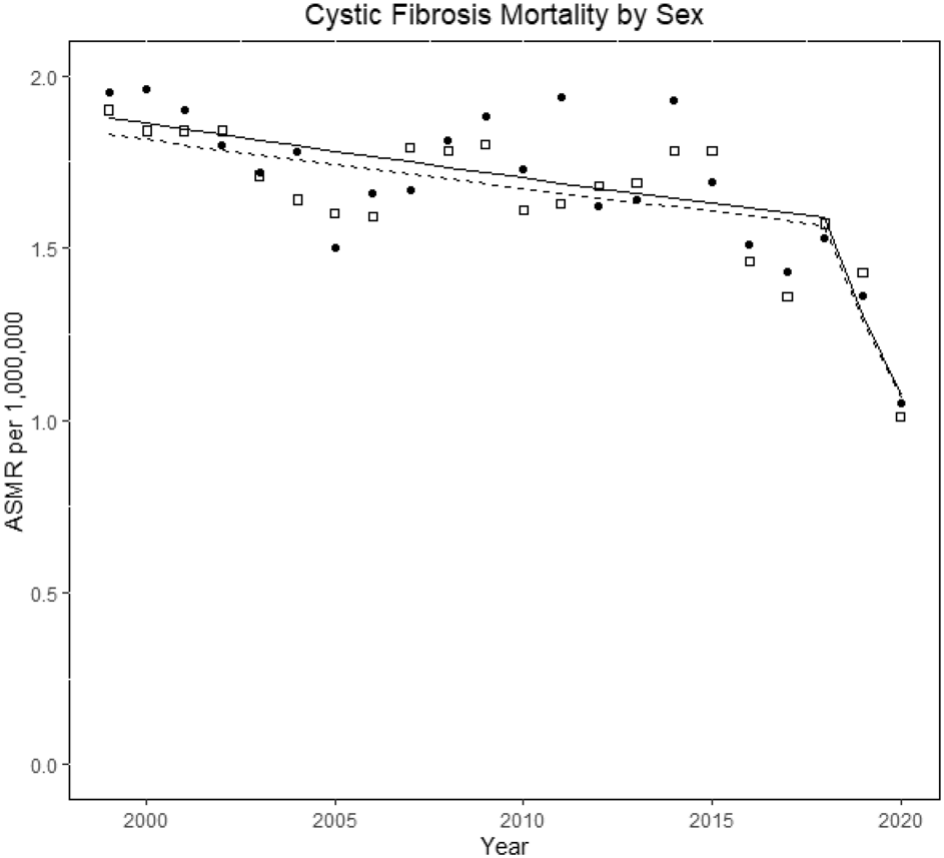
Joinpoint trends of cystic fibrosis-related age-standardized mortality rates per million population by sex in the United States, 1999–2020. Squares indicate men, whereas circles indicate women.

The median age of death was 24 years (interquartile range [IQR] 18–33) in 1999, and 37 years (IQR 25–55) in 2020 (Fig. 2). In 1999, a higher median age of death was observed in men compared to women, but the difference resolved over the study period (Table 3). The difference in the median age of death between Whites and other races slightly improved over the study period, but the gap widened between non-Hispanic and Hispanic ethnicities over time (Table 3).

**Table 3:** Median age of death in cystic fibrosis decedents in the US, 1999–2020.

	1st quartile	Median	3rd quartile	*p*-value: median difference
Years				< 0.01*
1999–2020	21	28	39	
1999	18	24	33	
2005	19	25	35	
2010	21	26	37	
2015	22	30	42	
2020	25	37	55	
Gender				< 0.01**
Men (1999–2020)	22	29	41	
Men (1999)	18.5	25	33	
Men (2020)	25	37	53	
Women (1999–2020)	20	27	38	
Women (1999)	17	23	32	
Women (2020)	25	37	55	
Race				< 0.01^^^
White (1999–2020)	21	28	40	
White (1999)	18	24	33	
White (2020)	26	37	55	
Others (1999–2020)	13	26	37	
Others (1999)	16	19	22	
Others (2020)	23	34	48	
Ethnicity				< 0.01†
Non-hispanic (1999–2020)	21	28	40	
Non-hispanic (1999)	13	24	**33**	
Non-Hispanic (2020)	26	38	59	
Hispanic (1999–2020)	14	22	31	
Hispanic (1999)	11	17	24	
Hispanic (2020)	20.5	26	49	

*P-value for median age of death comparing year 1999 to 2020; **Comparison between men (1999–2020) and women (1999–2020); ^^^Comparison between White (1999–2020) and Others (1999–2020); † Comparison between non-Hispanic (1999–2020) and Hispanic ethnicity (1999–2020).

*US* United States.

Data from multiple cause of death CDC WONDER data set, 1999 2020.

### Temporal joinpoint trends

**Figure 3:**
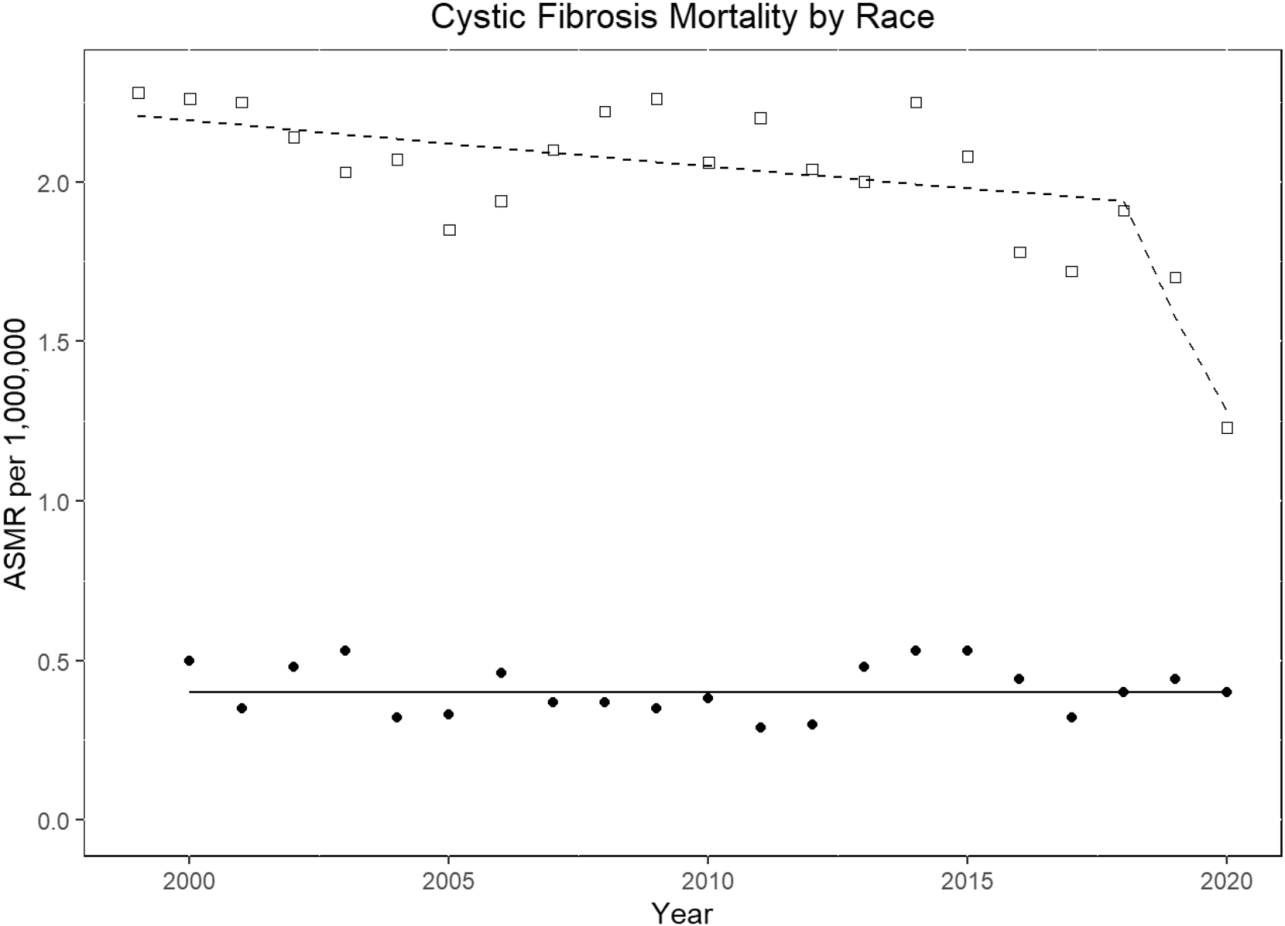
Joinpoint trends of cystic fibrosis-related age-standardized mortality rates per million population by race in the United States, 1999–2020. Squares indicate white, whereas circles indicate other races (African American, American Indians/Alaska Native, and Asian/Pacific Islanders).

In 1999, 539 individuals died from CF, and the annual number of deaths decreased to 342 in 2020. The corresponding CF ASMRs decreased from 1.9 per million population (CI 1.74–2.06) to 1.04 (CI 0.93–1.16) (AAPC, − 2.6% [CI − 4.7 to − 0.6]; *p* = 0.013). Two Joinpoint trends indicated a steady decline in mortality from 1999 to 2018 (EAPC, − 0.7% [CI, − 1.4 to 0]; *p* = 0.037), followed by a trend toward significant reduction from 2018 to 2020 (EAPC, − 19.1% [CI: − 35.7 to 1.8]; *p* = 0.068). Similar trends were observed individually in each sex (Tables 4, S1; Figs. 1, 3).

**Table 4:** Joinpoint temporal trends of cystic fibrosis-related age-standardized mortality rates per million population in the United States, 1999–2020.

Variables	Trend 1			Trend 2			Trend 3			Trend 4			Overall from 1999–2020	
	Years	EAPC (95% CI)	P value	Years	EAPC (95% CI)	P value	Years	EAPC (95% CI)	P value	Years	EAPC (95% CI)	P value	AAPC (95% CI)	P value
US overall	1999–2018	− 0.7 (-1.4–0)	0.037	2018–2020	− 19.1 (− 35.7 to 1.8)	0.068							− 2.6 (− 4.7 to − 0.6)	0.013
Gender														
Men	1999–2018	− 0.8 (− 1.4 to − 0.2)	0.013	2018–2020	− 17.6 (− 32.2 to 1.6)	0.067							− 2.6 (− 4.4 to − 0.7)	0.009
Women	1999–2018	− 0.9 (− 1.6 to − 0.2)	0.021	2018–2020	− 17.8 (− 35.9 to 5.4)	0.115							− 2.6 (− 4.8 to − 0.4)	0.022
Race														
White	1999–2018	− 0.7 (− 1.3 to 0)	0.043	2018–2020	− 18.8 (− 35 to 1.4)	0.064							− 2.6 (− 4.5 to − 0.6)	0.013
Non-white*	2000–2020	0 (− 1.5 to 1.5)	0.98										0 (− 1.5 to 1.5)	0.98
Ethnicity														
Non-Hispanic	1999–2018	− 0.9 (− 1.6 to − 0.2)	0.012	2018–2020	− 17.1 (− 34.1 to 4.3)	0.103							− 2.6 (− 4.6 to − 0.5)	0.016
Hispanic*	2000–2020	2.1 (0.6–3.6)	0.008										2.1 (0.6–3.6)	0.008
Census region														
Northeast	1999–2020	− 1.9 (− 2.8 to 1)	< 0.001										− 1.9 (− 2.8 to 1)	< 0.001
Midwest	1999–2015	− 0.4 (− 1.6 to 0.7)	0.445	2015–2020	− 8.7 (− 14.8 to − 2.2)	0.013							− 2.5 (− 4.2 to 0.8)	0.005
South	1999–2018	− 0.6 (− 1.2 to 0.1)	0.081	2018–2020	− 19 (− 35.3 to 1.4)	0.065							− 2.5 (− 4.5 to − 0.5)	0.016
West	1999–2020	− 0.9 (− 2.0 to 0.2)	0.096										− 0.9 (− 2.0 to 0.2)	0.096
Urbanization**														
> 250 K	1999–2018	− 1.2 (− 1.8 to − 0.6)	0.001	2018–2020	− 14.9 (− 30.7 to 4.5)	0.116							− 2.6 (− 4.4 to − 0.7)	0.007
< 250 K	1999–2015	0.9 (− 0.2 to 2.1)	0.095	2015–2020	− 9.9 (− 15.6 to − 3.8)	0.002							− 2.0 (− 3.2 to − 0.7)	0.002
Age (years)														
< 15	1999–2020	− 5.1 (− 6.0 to − 4.2)	< 0.001										− 5.1 (− 6.0 to − 4.2)	< 0.001
15–24	1999–2015	− 2.3 (− 3.3 to − 1.3)	< 0.001	2015–2020	− 14.6 (− 19.7 to − 9.2)								− 5.4 (− 6.8 to − 3.9)	< 0.001
25–34	1999–2005	− 3.9 (− 7.2 to − 0.5)	0.03	2005–2009	6.5 (− 4 to 18.2)	0.208	2009–2018	− 2.8 (− 5 to − 0.6)	0.019	2018–2020	0.01922.3 (− 36.9 to − 4.3)	0.022	− 3.5 (− 6.1 to − 0.8)	0.013
35–44	1999–2020	− 0.5 (− 1.6 to 0.7)	0.379										− 0.5 (− 1.6 to 0.7)	0.379
45–54	1999–2020	3.2 (2–4.3)	< 0.001										3.2 (2–4.3)	< 0.001
> 55	1999–2020	5.2 (3.7–6.8)	< 0.001										5.2 (3.7–6.8)	< 0.001
US overall^^^	1999–2018	− 1 (− 1.7 to 0.4)	0.004	2018–2020	− 22.1 (− 37.5 to 2.8)	0.029							− 3.2 (− 5.2 to 1.3)	< 0.001
US overall†	1999–2018	− 0.8 (− 1.5 to 0.2)	0.019	2018–2021	− 16.2 (− 25.2 to 6.1)	*0.004							− 3.1 (− 4.6 to 1.6)	< 0.001

*AAPC* Average annual percentage change, *ASMRs* Age-standardized mortality rates, *CI* Confidence interval, *EAPC* Estimated annual percentage change

*Data for year 1999 not available; **Urbanization > 250K = large to medium metropolitans; < 250K = small metro—non-metro; ^^^A temporal trends using underlying cause of death as case definition; and *†* Joinpoint analysis of CF related mortality from 1999 to 2021 using 2021 provisional MCOD data files.

Data from multiple cause of death CDC WONDER data set, 1999–2020.

**Figure 4:**
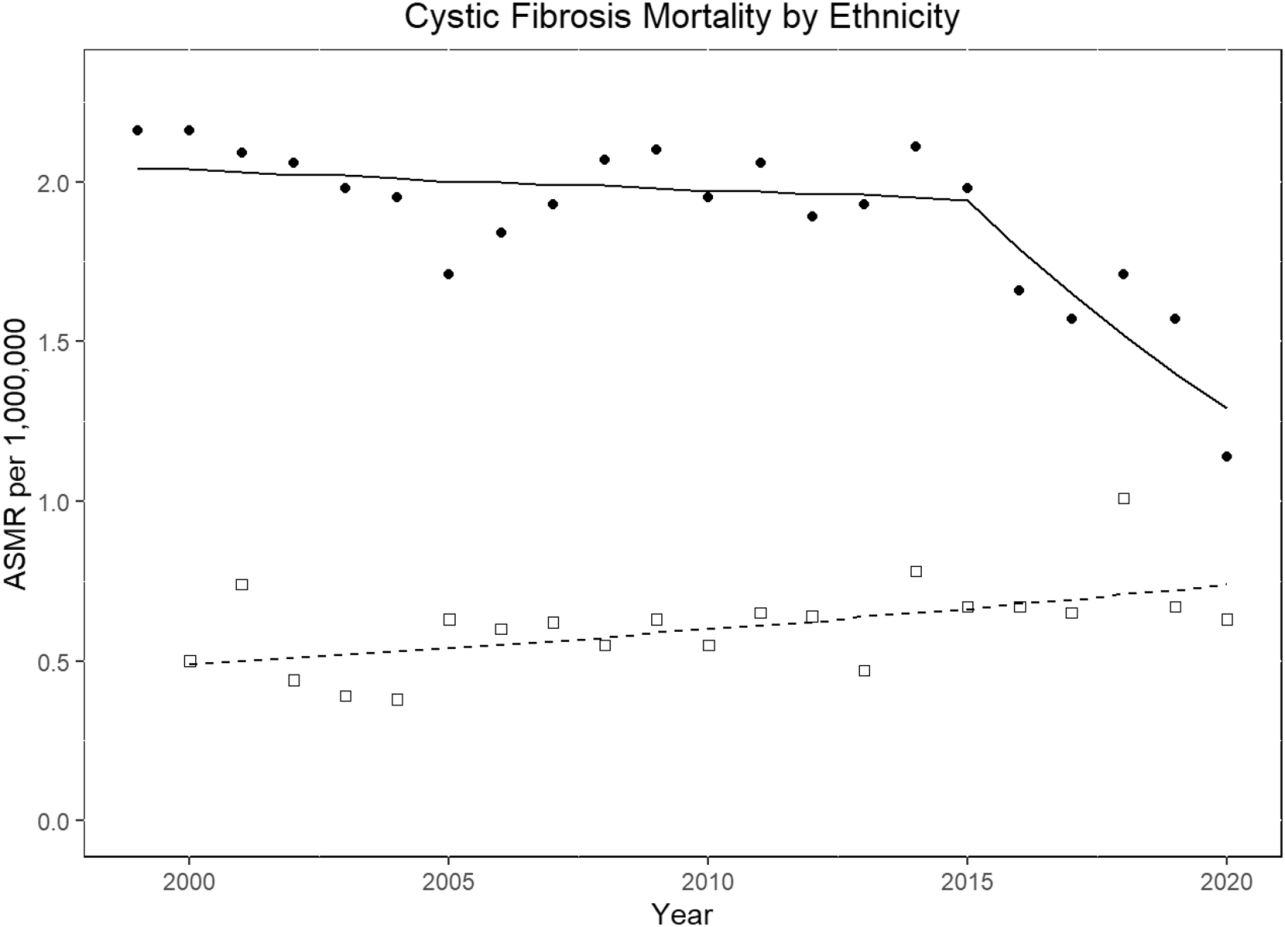
Joinpoint trends of cystic fibrosis-related age-standardized mortality rates per million population by ethnicity in the United States, 1999–2020. Squares indicate Hispanic, whereas circles indicate non-Hispanic population.

CF ASMRs decreased in the white population from 2.28 per million population (CI 2.09–2.48) to 1.23 (CI: 1.09–1.37) (AAPC: − 2.6% [− 4.5 to − 0.6]; *P* = 0.013). However, a flat trend was observed in all other races combined (AAPC, 0% [CI − 1.5 to 1.5]; *p* = 0.98) (Table 4; Fig. 3). In the non-Hispanics, ASMR decreased from 2.16 (CI 1.97–2.34) to 1.14 (CI 1–1.3) [AAPC, − 2.6% (CI − 4.6 to − 0.5); *p* = 0.016]. In the Hispanic cohort, ASMR increased from 0.5 (CI 0.31–0.78) in 2000 to 0.63 (CI 0.44–0.89) in 2020 (AAPC 2.1% [CI 0.6–3.6]; *p* = 0.008) (Table 4; Fig. 4). The CF mortality decreased in all the census regions (*p* < 0.05) except West (*p* = 0.096) (Table 4). CF mortality rates decreased irrespective of urbanization status (*p* < 0.05) (Table 4).

In 1999, the highest CF mortality was seen in the 15–24 years age group [5.09 per million population (CI 4.38–5.80)] and the lowest mortality was observed in > 55-years age group [0.34 (CI 0.21–0.53)]. The CF mortality improved in < 35 years age groups (*p* < 0.05). There was no change in mortality in the 35–44 years age group (*p* = 0.379). Conversely, an increase in mortality was recorded in > 45-year age groups (*p* < 0.001) (Table 3).

The CF-related mortality was reanalyzed using UCOD as the case definition. The ASMR decreased from 1.71 per million population (CI 1.56–1.87) in 1999 to 0.82 (CI 0.72–0.92) in 2020 (AAPC, − 3.2% [CI − 5.2 to − 1.3%]; *p* < 0.001). Again, two Joinpoint trends were observed (Tables 4, S1 and S2). Notably, out of 342 CF-related deaths in 2020, only 6 (1.7%) had SARS-CoV-2 as the UCOD.

### Sensitivity analysis

The first post hoc sensitivity analyses with provisional data from 2021 demonstrated a decrease in deaths from 342 in 2020 to 314 in 2021. The corresponding ASMRs decreased from 1.04 per million population (CI 0.93–1.16) to 0.95 (CI 0.85–1.06) (AAPC, − 3.2% [CI − 5.2 to − 1.3]; *p* < 0.01). Similar to the primary analysis, the Joinpoint analysis showed two trends; 1999–2018 (EAPC, − 0.8% [CI − 1.5 to − 0.2]; *p* = 0.019) and 2018–2021 (EAPC, − 16.2% [CI − 25.2 to − 6.1); *p* = 0.004], suggesting the durability of the significant decrease in CF-related mortality following 2018 and beyond 2020 (Fig. S2). In the second post hoc analysis, the mortality trends of chronic respiratory diseases showed an increase from 84.13 (CI 83.85–84.41) in 2019 to 92.22 (CI 91.93–92.51) in 2020 which was incongruent with CF-related ASMRs in the same period.

## Discussion

There has been a significant decline in CF-related mortality in the US. While there were only gradual improvements in the earlier part of the past two decades, there has been a rapid decline in recent years. The median age of death has increased by 13 years, and the mortality rates decreased consistently across sex, white race, non-Hispanic ethnicity, census regions, and urbanization status. Conversely, the mortality rates worsened in Hispanic ethnicity and persisted without change in non-white races. In parallel, significantly lower median age of death was observed in Hispanic ethnicity and non-white races, associated with a widening gap with their respective counterparts over time. It is also worth noting that men and women had similar overall ASMRs, but women have higher mortality rates from 1 to 24 years of age and a lower median age of death, indicating a relatively poor prognosis in younger females.

The temporal trends of CF-related mortality from 1979 through 1991 were reported using the US CDC data files^12^, and our work builds upon this previous study. Halliburton et al.^12^ reported a 21% decrease in CF-ASMR and an increase in the median age of death among CF decedents from 15 to 23 years. Our analysis demonstrated a 45% decrease in CF-ASMR and an increase in the median age of death from 24 to 37 years. Consistent with our findings, the CFFPR annual report documented an improvement in the mortality rate from 1999 to 2020, although the magnitude of change was relatively higher at 62%^8^. Stephenson and colleagues also reported improved survival in the US and Canada; however, there was a notably lower median age of survival in the US CF population, with an absolute difference of 10 years compared to their Canadian counterparts^10^. Of note, our analysis identified 1107 CF-related deaths from 2019 to 2021, whereas the CFFPR annual report^8^ reported a relatively lower number of deaths (n = 861), indicating some degree of selection bias in registry-based mortality studies and underscoring the inclusive nature of the current analysis.

Due to the observational nature of this data, we are unable to make causal statements about the exact causes of the significant decrease in CF-related mortality recently. However, a potential contributor to the decreasing CF mortality could be the introduction of CFTR modulator therapy. Ivacaftor, the first CFTR modulator, received approval in 2012 and treated only 4–6% of the CF population^15^. Subsequently, the introduction of Lumacaftor-ivacaftor in July 2015 expanded the coverage to 25–30% of the CF population^15^. With the introduction of tezacaftor-ivacaftor in February 2018, the coverage further increased to 45% of CF patients. In October 2019, triple modulator therapy was approved for those with the F508del mutation, covering 90% of the CF population^8,15^. The introduction of the early access program for triple modulator therapy in the summer of 2019 for those with the most advanced disease led to the fast uptake of triple modulator therapy in the US. Out of all the eligible patients for any modulator therapy, 25% were on triple therapy in 2019, 63% in 2020, and 76% in 2021^8,16^. Our analysis revealed a parallel trend between the stepwise approval of CFTR modulators and an increase in the median age of death. Specifically, from 2011 to 2015, there was a 4-year increase, and from 2016 to 2020, there was a 7-year increase, compared to 2 years in the previous decade. Additionally, the introduction of triple modulator therapy correlated with a fast decrease of 26% in CF-ASMR in 2020. The provisional CDC MCOD data files showed a continuous but relatively slower drop of 9% in 2021. These findings align with the mortality trends reported by the CFFPR in their annual reports^8,16^, although the magnitude of mortality rate reduction recorded by the CFFPR annual reports was higher at 33.3% and 13% in 2020 and 2021, respectively. Notably, the decrease in mortality burden documented in the CFFPR reports was predominantly related to the non-lung transplant CF population^8,16^. Another possible explanation could be the onset of the SARS-CoV-2 pandemic which coincided with the beginning of the fast decrease in CF mortality since 2019. We conducted a post hoc sensitivity analysis of national mortality trends in other common chronic respiratory diseases, which may mimic the CF population but did not observe similar trends in those populations. Interestingly, we found that SARS-CoV-2 infection was identified as a contributing cause of death in only 8 (2.3%) CF decedents in 2020.

Historically, studies have reported a lower median age of death or median survival age in CF females compared to males. The current analysis found a similar disparity at the beginning of the study but a complete closure of the gap in the year 2020, with the same median age of death between genders, which is a novel finding. Previous studies from the 1990s indicated lower survival age in CF females^17,18^. In a study by Rosenfield et al. in 1997^19^, the median survival age was reported as 25.3 years in females and 28.4 years in males, using the CFFPR database. The exact reasons for this disparity are not yet fully understood. Demko et al.^20^ observed that CF females tend to acquire Pseudomonas aeruginosa infection at an earlier age and experience a faster decline in forced expiratory volume in 1 s (FEV1). Estrogen has been implicated in modulating ion channels in the airway epithelium, leading to increased mucus viscosity and promoting the transition of non-mucoid Pseudomonas aeruginosa infection to the mucoid type, which is a drug-resistant subtype associated with a poorer prognosis^21,22^. In 2014, Harness-Brumley et al.^23^ conducted a large cohort analysis using the CFFPR database, involving 32,766 patients over a 13-year period, and reported persistent differences in life expectancy between males and females. The authors also found that females become colonized with various microorganisms such as Pseudomonas aeruginosa, Staphylococcus aureus, Hemophilus influenzae, Aspergillus, and non-tuberculous mycobacterium at earlier ages^23^. Possible explanations for the closing of the mortality gender gap are the modern aggressive treatment of CF, including the use of newer antipseudomonal antimicrobials, DNase therapy to improve mucus clearance, and most recently, the highly effective CFTR modulators. Furthermore, there has been increased standardization of care for CF across the country, with specialized CF care centers playing a crucial role.

To our knowledge, this is the first nationwide US study that explores the temporal trends of CF-related mortality by ethnicity over a period of two decades. In 1999, the median age of death among Hispanic CF decedents was 17 years, compared to 24 years in non-Hispanic individuals, indicating poorer survival in the former. Over the study period, the gap between the two ethnicities widened from 7 to 12 years, and the median age of death increased to 26 years in Hispanics and 38 years in non-Hispanics in 2020. The Joinpoint analysis also revealed divergent mortality trends between the two ethnic groups, indicating a worsening of inequalities over time. A study by Rho et al.^24^ documented a lower mean age of death among Hispanic CF decedents from 2010 to 2014, but the gap between the ethnicities was relatively smaller. Two earlier studies conducted from 1982 to 1998 and 1991 to 2010 reported up to three times the risk of death among Hispanic CF individuals, even after adjusting for socioeconomic factors^25,26^. The exact reasons for the relatively poor outcomes in the Hispanic CF population could not be determined from our analysis. Possible explanations, extrapolated from other diseases, include lower health literacy, lower medical insurance coverage^27^, language barriers, treatment non-adherence^28,29^, higher exposure to tobacco or air pollution^30–33^, cultural acculturation^34^, and higher rates of malnutrition^35^.

Other factors that have been identified include older age at diagnosis^33^ and differential access to lung transplantation^36,37^. Hispanic patients tend to acquire Pseudomonas infection more frequently and at an earlier age, have lower FEV1, and are underrepresented in clinical trials^34,37–40^. The differences in mortality may also be influenced by variations in the identification of genetic mutations, with 39% of CFTR variants in Hispanic CF individuals remaining unclassified^33,41,42^. Moreover, the heterogeneity in CFTR genotype distribution results in 25% of Hispanic CF patients currently being ineligible for available CFTR modulator therapy based on their genotypes, compared to 10% in non-Hispanic whites^26^. This relative ineligibility for CFTR modulator therapy may further contribute to the survival differences^43^.

There is a wealth of literature reporting higher morbidity and mortality in rural areas, termed "rural penalty mortality"^44,45^. The rural–urban divide for chronic lower respiratory diseases such as COPD and asthma has been reported, with worsening inequalities over time^46^. We believe that this is the first study exploring CF-related mortality by urbanization status. We found a higher CF-ASMR in the rural population; however, the rural–urban divide has narrowed in recent years due to improvements in rural population mortality. Potential reasons for these inequalities may include fewer specialized CF care centers in smaller counties, different smoking rates, and socioeconomic factors. Future studies focused on exploring the key factors responsible for these disparities are required to identify areas for intervention.

Cystic fibrosis patients frequently die young from the complications of the disease, while common geriatric diseases contribute less^8,9,47^. Recent advances in CF management have led to a step-change in survival. Studies on the impact of recent advances in CF therapies predict a survival age of 56 years and 66 years for individuals born in 2010 and 2021, respectively^8,48^. With the increasing survival, the number of adult CF patients is estimated to increase by 75% from 2015 through 2025^49^. The rapidly changing epidemiology of CF presents new challenges, requiring different and more collaborative approaches to healthcare delivery to meet future needs adequately. The increased risk of gastrointestinal malignancies^50^ and complications associated with long-standing CF-related diabetes underscore the importance of surveillance and interventional care in the coming decades. This will necessitate more active partnerships with primary care physicians, as well as cardiovascular, endocrine, and gastrointestinal specialists^51,52^.

Our study is observational, and there are several inherent limitations to consider. Although the CDC WONDER database provides a comprehensive assessment of mortality statistics, it relies on information from death certificates, which may be prone to reporting errors or misclassification of the cause of death^53,54^. Centralized data sources have been reported to underestimate mortality^55,56^. Additionally, not all individuals with CF may have CF documented as a contributing cause of death on their death certificates. It is important to note that the mortality estimates analyzed by the CDC do not account for changes in the incidence or prevalence of the disease. To address this limitation, we reported the median age of death as an additional surrogate marker of survival to assess temporal trends. However, it is pertinent to note that the median age of death reflects the age distribution of the decedents in a given year and is not analogous to the survival age of the entire CF population. Finally, due to the observational nature of our study and the unavailability of CFTR modulator treatment data, the results cannot be used to establish causality.

## Conclusions

The national CF-related mortality rates have declined, and the median age of death among CF decedents has significantly increased, indicating improved survival. These changes were relatively slow during an earlier period of the study, followed by a rapid decline more recently. We observed patterns of disparities based on sex, ethnicity, race, and geographical factors. These disparities were associated with a widening gap between ethnicities, a narrowing gap between races and rural vs. urban counties, and a complete closer of the gap between sexes over the study period.

### Supplementary Information

Supplementary Legends.

Supplementary Figure S1.

Supplementary Figure S2.

Supplementary Table S1.

Supplementary Table S2.

## Data Availability

The datasets generated and/or analyzed during the current study are available in the CDC WONDER repository, [https://wonder.cdc.gov/controller/datarequest/D77].

## Abbreviations

AAPC: Average annual percentage change
ASMR: Age-standardized mortality rate
CDC WONDER: Centre for Disease Control and Prevention Wide-ranging Online Data for Epidemiologic Research
CF: Cystic fibrosis
CFFPR: Cystic Fibrosis Foundation Patient Registry
CFTR: Cystic fibrosis transmembrane conductance regulator
CI: Confidence interval
EAPC: Estimated annual percentage change
ICD-10: International Classification of Diseases-10
IQR: Interquartile range
MCOD: Multiple cause of death
NCHS: National Center for Health Statistics
PC: Percentage change
SARS-CoV2: Severe acute respiratory syndrome coronavirus 2
UCOD: Underlying cause of death
US: United States
