# Supplementary material for: Cystic fibrosis-related mortality in the United States from 1999 to 2020: an observational analysis of time trends and disparities
Source: Sci Rep. 2023 Sep 12;13:15030. doi: 10.1038/s41598-023-41868-x (PMC10497589; doi:10.1038/s41598-023-41868-x)
Supplement: Supplementary file 3 — Supplementary Figure S2. [file 41598_2023_41868_MOESM3_ESM.docx]

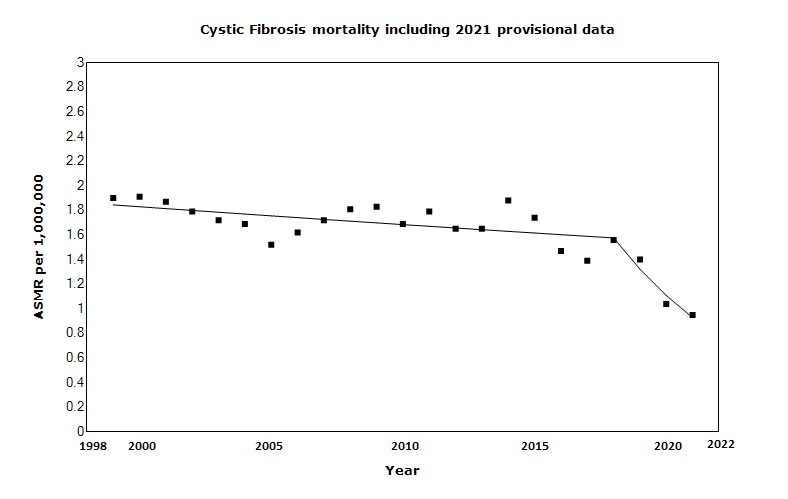


Figure S2: Joinpoint trends of cystic fibrosis-related age-standardized mortality rates per million population including provisional data from year 2021 in the United States, 1999-2021.
