# Supplementary material for: Cystic fibrosis-related mortality in the United States from 1999 to 2020: an observational analysis of time trends and disparities
Source: Sci Rep. 2023 Sep 12;13:15030. doi: 10.1038/s41598-023-41868-x (PMC10497589; doi:10.1038/s41598-023-41868-x)
Supplement: Supplementary file 4 — Supplementary Table S1. [file 41598_2023_41868_MOESM4_ESM.docx]

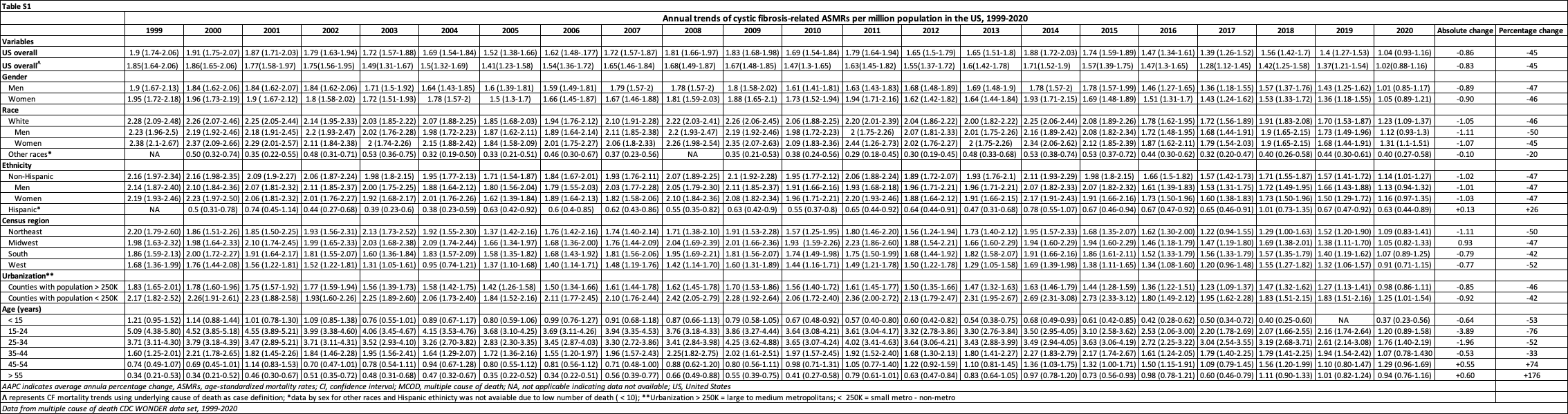


Table S1: Annual cystic fibrosis-related age-standardized mortality rates per million population in the United States, 1999-2020.
