# Supplementary material for: Cystic fibrosis-related mortality in the United States from 1999 to 2020: an observational analysis of time trends and disparities
Source: Sci Rep. 2023 Sep 12;13:15030. doi: 10.1038/s41598-023-41868-x (PMC10497589; doi:10.1038/s41598-023-41868-x)
Supplement: Supplementary file 5 — Supplementary Table S2. [file 41598_2023_41868_MOESM5_ESM.docx]

Table S2: Cystic fibrosis-related average age-standardized mortality rates per million population using underlying cause of death as case definition in the United States, 1999-2020.
